# Human Polyclonal Antibodies Prevent Lethal Zika Virus Infection in Mice

**DOI:** 10.1038/s41598-019-46291-9

**Published:** 2019-07-08

**Authors:** Emilie Branche, Ayo Yila Simon, Nicholas Sheets, Kenneth Kim, Douglas Barker, Anh-Viet T. Nguyen, Harpreet Sahota, Matthew Perry Young, Rebecca Salgado, Anila Mamidi, Karla M. Viramontes, Trevor Carnelley, Hongyu Qiu, Annie Elong Ngono, Jose Angel Regla-Nava, Mercylia Xevana Susantono, Joan M. Valls Cuevas, Kieron Kennedy, Shantha Kodihalli, Sujan Shresta

**Affiliations:** 1 La Jolla Institute for Immunology 9420 Athena Circle, La Jolla, CA 92037 USA; 2 Research and Development, Emergent BioSolutions Canada Inc, 155 Innovation Drive, Winnipeg, MB R3T 5Y3 Canada; 3 Medical Affairs, Emergent BioSolutions Canada Inc, 155 Innovation Drive, Winnipeg, MB R3T 5Y3 Canada

**Keywords:** Protein vaccines, Viral infection

## Abstract

Zika virus (ZIKV) is an emerging mosquito-borne flavivirus that represents a major threat to global health. ZIKV infections in adults are generally asymptomatic or present with mild symptoms. However, recent outbreaks of ZIKV have revealed that it can cause Congenital Zika Syndrome in neonates and Guillain-Barré syndrome in adults. Currently, no ZIKV-specific vaccines or antiviral treatments are available. In this study, we tested the efficacy of convalescent plasma IgG hyperimmune product (ZIKV-IG) isolated from individuals with high neutralizing anti-ZIKV titers as a therapeutic candidate against ZIKV infection using a model of ZIKV infection in *Ifnar1*^−/−^ mice. ZIKV-IG successfully protected mice from lethal ZIKV challenge. In particular, ZIKV-IG treatment at 24 hours after lethal ZIKV infection improved survival by reducing weight loss and tissue viral burden and improving clinical score. Additionally, ZIKV-IG eliminated ZIKV-induced tissue damage and inflammation in the brain and liver. These results indicate that ZIKV-IG is efficacious against ZIKV, suggesting this human polyclonal antibody is a viable candidate for further development as a treatment against human ZIKV infection.

## Introduction

Zika virus (ZIKV) is an arthropod-borne virus belonging to the family *Flaviviridae*, similar to dengue, West Nile, Japanese encephalitis, and yellow fever viruses^1^. ZIKV was first identified in a sentinel rhesus monkey in the Zika Forest of Uganda in 1947 and was isolated from mosquitoes (*Aedes africanus*) in 1948^2^. ZIKV is transmitted through the bite of infected female *Ae. aegypti* mosquitoes^3^ and potentially *Ae. albopictus* mosquitoes^4^, as well as alternative non-vector routes which have been identified, including vertical (i.e., mother-to-infant)^5–8^, transfusion^9–11^, and sexual transmission^12^. From the 1950’s to 1990’s, serological evidence of ZIKV was reported in multiple Asian^13–16^ and African^17–22^ countries, but no outbreaks and only 14 cases of human ZIKV disease were described^17,22–25^. The first ZIKV outbreak was observed in 2007 on Yap Island in the Federated States of Micronesia^26^, followed by a second outbreak in French Polynesia in 2013^27^. The most recent reported outbreak was on a larger scale that occurred from 2014 to 2016 in Latin America^28–30^. Interest in this virus increased after these outbreaks in part due to the emergence of ZIKV outside its previously known geographic range, showing the potential of the virus to spread wherever the mosquito vector is present. In addition, prior to the French Polynesia outbreak, ZIKV was known to be asymptomatic or cause only mild symptoms (fever, headache, malaise, arthralgia, myalgia, maculopapular rashes, and conjunctivitis). However, since 2007, severe complications of ZIKV infection, in particular Guillain–Barré Syndrome in adults^31,32^ and Congenital Zika Syndrome in babies born to ZIKV-infected women^7,8,33–36^ have been observed. These findings led the WHO to declare ZIKV a public health emergency of international concern in 2016 and expanded efforts for the development of vaccines and therapeutics to combat the disease.

Antibodies (Abs) have been shown to play a critical role in the protective immune response against infectious diseases and have been used for passive immunization, in the prevention and treatment of both bacterial and viral infections, for more than a century. Immune animal sera were first used in the late 1800’s for treatment of disease, followed by an era of immune human serum therapy for both viral and bacterial diseases. Notably, during the 1918 influenza pandemic, serum from recovered patients was used successfully to treat acutely ill patients^37^. The role of convalescent serum therapy expanded to many infections beyond influenza during the first half of the 20th century with clinical benefit demonstrated for other viral diseases like measles^38^ and polio^39^, and for invasive bacterial pathogens, including pneumococcus, *Haemophilus influenzae B*, and meningococcus^40,41^. Passive immunization with antibody-based therapies has emerged as a promising strategy for treating emerging infectious diseases, and include both monoclonal (mAb) and polyclonal antibodies (pAb), each of which has its advantages and disadvantages. For example, mAbs can be easily manufactured in large quantities and have a greater inherent biological consistency due to their epitope specificity as compared to pAbs. However, mAbs have limitations, including development of escape mutants and high production costs. In comparison with mAbs, pAbs can have more robust activities, neutralizing several virus strains even after viral mutations^42,43^. Although several studies have demonstrated that mAbs can provide therapeutic protection against ZIKV in various human and mouse models^44^, only a single study to date has shown therapeutic potential of human pAbs produced from transchromosomal cows against ZIKV infection in mice. As no study has as yet assessed the efficacy of pAbs isolated from humans as a potential therapeutic against ZIKV, herein, we evaluate the therapeutic potential of a pAb preparation from human plasma containing high anti-ZIKV titers (ZIKV-IG).

Specifically, we used the *Ifnar1*^−/−^ mouse model as a stringent challenge system to evaluate the therapeutic potential of ZIKV-IG. Prior to the recent ZIKV epidemics, only a few studies had been performed in mice and these required many serial passages of ZIKV in mice to produce consistent disease phenotypes^45–47^. Within the last three years, substantial efforts have been focused on generating new mouse models. ZIKV evades the anti-viral type I interferon (IFN) response, in part through inhibition of the STAT2 and STING pathways in human but not mouse cells^48–50^. This antagonism of the type I IFN receptor (Ifnar) signaling in a species-specific manner by ZIKV explains the more severe pathogenesis of ZIKV infection in mice with immature or compromised immune systems compared to adult immunocompetent mice, and why disruption of the Ifnar1 signaling increases susceptibility of mice to lethal ZIKV infection. Accordingly, wild type mice treated with blocking anti-*Ifnar1* mAb^51–54^, and mice gene-deficient for *Ifnar1*^53–58^ or for both *Ifnar1* and type II IFN receptors^59–61^ have been widely used as models of ZIKV infection. We measured the effectiveness of ZIKV-IG therapy on survival, viral burden and tissue pathology in key organs, including spleen, kidneys, liver, sciatic nerves and brain, of *Ifnar1*^−/−^ mice following lethal ZIKV challenge. ZIKV-IG treatment at 24 hrs post-infection increased survival by reducing viral burden and ZIKV-induced tissue damage and inflammation in several key organs. These findings demonstrated that a single dose of ZIKV-IG is efficacious against lethal Zika disease in a highly stringent mouse challenge model.

## Results

### ZIKV-IG decreases morbidity and mortality in a dose dependent manner in *Ifnar1*^−/−^ mice

**Figure 1:**
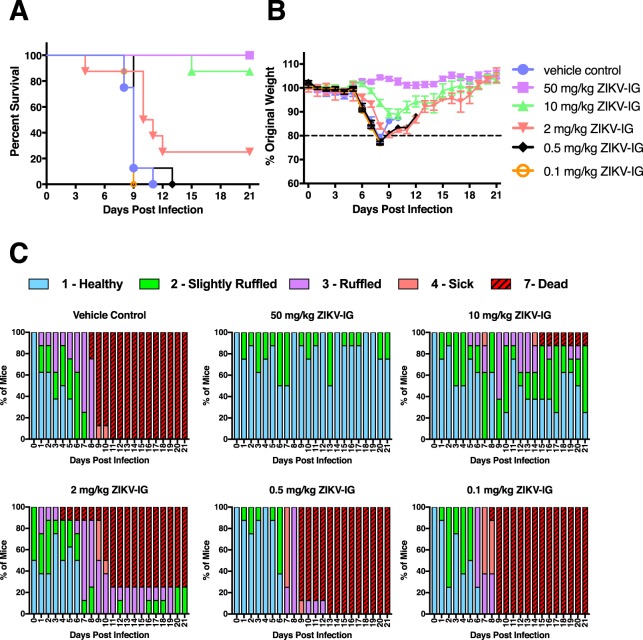
ZIKV-IG treatment improves survival of ZIKV-infected mice. Groups of *Ifnar1*^−/−^ mice (n = 8) were infected with 1.0 × 10^3^ FFU of ZIKV strain FSS13025 via a retro-orbital (r.o.) route. At 24 hrs p.i., mice were treated (via r.o. route) with vehicle, 50, 10, 2, 0.5 or 0.1 mg/kg ZIKV-IG. (**A**) Kaplan–Meier survival curves. (**B**) Mean percent weights, which are plotted for each group relative to the percent weight on day 0 (baseline). (**C**) Clinical scores. Error bars represent standard error of the mean.

To evaluate the therapeutic efficacy of ZIKV-IG against ZIKV infection, *Ifnar1*^−/−^ mice were infected with ZIKV (strain FSS13025, 1.0 × 10^3^ FFU/mouse, retro-orbital (r.o.) route) and then treated with ZIKV-IG (50, 10, 2, 0.5 and 0.1 mg/kg, r.o. route) 24 hrs post-infection (p.i.). ZIKV-IG used for this study exhibits high neutralization activity against multiple ZIKV strains, including strain FSS13025 (Supplementary Fig. S1). Animals were observed daily for survival, body weight changes and clinical signs of disease for day 21 p.i. For the entire study, vehicle control has been used as a negative control because the outcomes for animals treated with vehicle control were not significantly different to those observed in animals treated with a naïve-ZIKV-IG placebo, and significant differences were observed between 50 mg/kg ZIKV-IG treated mice and both vehicle control (p = 0.0022) and naïve-ZIKV-IG (p = 0.0022) treated mice (Supplementary Fig. S2 and Table S1). A dose-dependent effect on mortality, weight changes and severity of ZIKV disease was observed in ZIKV-IG treated animals compared to those treated with vehicle control (Fig. 1). Specifically, mice treated with the highest dose of ZIKV-IG (50 mg/kg) displayed a 100% survival rate, which was significantly greater than controls (0% survival, p = 0.0005), and similarly high survival rate was observed in mice treated with 10 mg/kg as compared to control mice (87.5% survival, p = 0.0042). Survival rates in the lower dose groups of 25%, 0%, and 0% (2, 0.5 and 0.1 mg/kg, respectively) were not statistically different than for control animals (Fig. 1A and Table 1). Similar patterns were observed in analysis of median time to death, with statistically significant increases in median time to death compared to controls for the 50 mg/kg and 10 mg/kg dose groups (p = 0.0001 and p = 0.0008, respectively) but not for the lower dose groups (Table 2). As with mortality, the 50 mg/kg dose group showed reduced morbidity compared to the control group, with significantly different weight change and clinical score outcomes over the 21-day observation period (Fig. 1B,C). The 50 mg/kg dose group exhibited little to no weight loss and clinical signs of disease, while the 10 mg/kg dose group showed reduced morbidity compared to controls, but only in terms of clinical score outcomes (Fig. 1C) and not in terms of body weight (Fig. 1B). In contrast, the lowest dose groups ( ≤ 2 mg/kg) exhibited progressive deterioration of weight (Fig. 1B) and clinical score (Fig. 1C) beginning at day 6 p.i. and extending to time of death or severe disease necessitating euthanasia. These data indicate that the 50 mg/kg dose is the most efficacious of the doses tested against ZIKV infection in this mouse model.

**Table 1:** Analysis of survival rate between vehicle control group and ZIKV-IG-treated groups.

Comparison to vehicle control			
Treatment Group	% Survival	Fisher’s Exact Test p-value	Bonferroni Adjusted p-value
Vehicle control	0 (0/8)	NA	NA
50 mg/kg ZIKV-IG	100 (8/8)	0.00016**	0.0005**
10 mg/kg ZIKV-IG	87.5 (7/8)	0.00014**	0.0042**
2 mg/kg ZIKV-IG	25 (2/8)	0.467	1.000
0.5 mg/kg ZIKV-IG	0 (0/8)	NA	NA
0.1 mg/kg ZIKV-IG	0 (0/8)	NA	NA

**Table 2:** Analysis of median time to death between vehicle control group and ZIKV-IG-treated groups.

Comparison to vehicle control			
Treatment Group	Median Survival (days)	Logrank Test p-value	Bonferroni Adjusted p-value
Vehicle control	9	NA	NA
50 mg/kg ZIKV-IG	Undefined	<0.0001***	0.0001***
10 mg/kg ZIKV-IG	Undefined	<0.0001***	0.0008**
2 mg/kg ZIKV-IG	10.5	0.126	1.000
0.5 mg/kg ZIKV-IG	9	0.675	1.000
0.1 mg/kg ZIKV-IG	9	0.919	1.000

### ZIKV-IG treatment decreases viral replication and dissemination in *Ifnar1*^−/−^ mice

**Figure 2:**
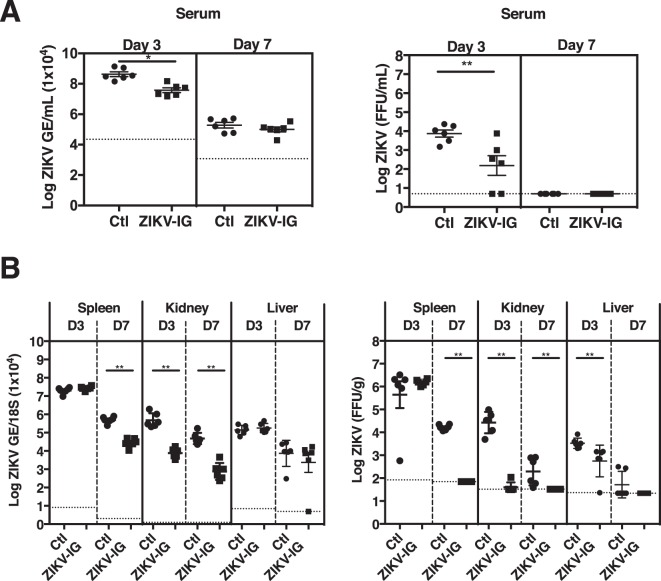
ZIKV-IG treatment decreases viral burden in key non-neuronal target organs of ZIKV in mice. Groups of *Ifnar1*^−/−^ mice (n = 6) were infected with 1.0 × 10^3^ FFU of ZIKV strain FSS13025 (via r.o. route). At 24 hrs p.i., mice were treated (r.o. route) with either vehicle (circles) or 50 mg/kg ZIKV-IG (squares). Viral RNA and infectious viral particle levels were determined by qRT-PCR and FFA, respectively, at days 3 and 7 p.i. in the (**A**) serum and (**B**) kidney, spleen and liver. Dotted lines indicate the limit of detection. The p values were obtained using non-parametric Wilcoxon Rank-Sum tests followed by the Bonferroni correction. Study groups were compared for statistical significance for each tissue and time-point. Error bars represent the standard error of the mean.

To determine how ZIKV-IG treatment improves morbidity and mortality, we examined whether ZIKV-IG treatment reduces viral burden in sera and key target organs of ZIKV tropism (spleen, kidneys, liver, sciatic nerves, and brain). *Ifnar1*^−/−^ mice were infected with ZIKV (strain FSS13025, 1.0 × 10^3^ FFU/mouse, r.o. route) followed by treatment at 24 hrs p.i. with ZIKV-IG (50, 10, 2 and 0.5 mg/kg, r.o. route). Sera and organs were harvested on days 3 and 7 p.i., and levels of viral RNA and infectious virus to BHK cells were determined by qRT-PCR and FFA, respectively. The 0.1 mg/kg dose was not used in this experiment due to the observed similarities of mortality and morbidity between this group and the 0.5 mg/kg group. In the serum, only 50 mg/kg ZIKV-IG-treated mice had significant reductions in both viral RNA (−1.1-fold, p = 0.017) and infectious virus levels (−1.8-fold, p = 0.043) at day 3 p.i. relative to vehicle-treated control mice (Fig. 2A and Supplementary Table S2). No significant reductions were noted in any animals treated with ≤10 mg/kg ZIKV-IG on day 3 p.i. or treated with any dose level on day 7 p.i. compared to controls, suggesting that by day 7 p.i. virus is cleared from circulation in all groups (Supplementary Fig. S3 and Table S2). In the spleen, similar levels of viral RNA and BHK cell-infectious viral particles were present in all groups at day 3 p.i., but at day 7 p.i., animals treated with 50 mg/kg ZIKV-IG had lower levels of viral RNA (−1.3-fold, p = 0.009) and BHK cell-infectious virus (−2.3-fold, p = 0.009) relative to vehicle-treated control mice (Fig. 2B and Supplementary Table S2). Spleens of mice treated with 10 mg/kg ZIKV-IG (−1.3-fold, p = 0.009 for viral RNA and −1.7-fold, p = 0.009 for BHK cell-infectious ZIKV) and 2 mg/kg ZIKV-IG (−1.1-fold, p = 0.017 for viral RNA and −1.3-fold, p = 0.035 for infectious ZIKV) also contained lower levels of viral RNA and BHK cell-infectious virus than control mice at day 7 p.i. (Supplementary Fig. S3 and Table S2). In the kidney, 50 mg/kg ZIKV-IG treatment significantly reduced levels of viral RNA and BHK cell-infectious virus compared to controls at both day 3 p.i (−1.5-fold, p = 0.009 for viral RNA; −2.8-fold, p = 0.009 for infectious virus) and day 7 p.i. (−1.6-fold, p = 0.009 for viral RNA; −1.5-fold, p = 0.009 for infectious ZIKV) (Fig. 2B and Supplementary Table S2). Kidneys in animals treated with 10 mg/kg ZIKV-IG also contained decreased levels of viral RNA relative to control mice at day 3 p.i. (−1.1-fold, p = 0.035) (Supplementary Fig. S3 and Table S2). In the liver, 50 mg/kg ZIKV-IG-treated mice had similar levels of viral RNA but lower levels of BHK cell-infectious ZIKV (−1.3-fold, p = 0.009) than control mice at day 3 p.i. (Fig. 2B and Supplementary Table S2). At day 7 p.i., the liver RNA levels were significantly elevated in mice treated with 10 mg/kg (1.3-fold, p = 0.009), 2 mg/kg (1.5-fold, p = 0.009) and 0.5 mg/kg ZIKV-IG (1.3-fold, p = 0.017) as compared to control mice (Supplementary Fig. S3 and Table S2); however, no difference in levels of BHK cell-infectious virus was observed between the liver from any of the ZIKV-IG treated groups and control mice at day 7 p.i. (Supplementary Fig. S3 and Table S2). The increased viral RNA levels observed in the liver could be related to the analytical methods, as qRT-PCR detects viral RNA of both infectious and non-infectious virus particles, whereas the FFA analysis detects only BHK cell-infectious virus particles. Collectively, these results demonstrate that ZIKV-IG treatment reduces viral burden in key non-neuronal target tissues of ZIKV.

**Figure 3:**
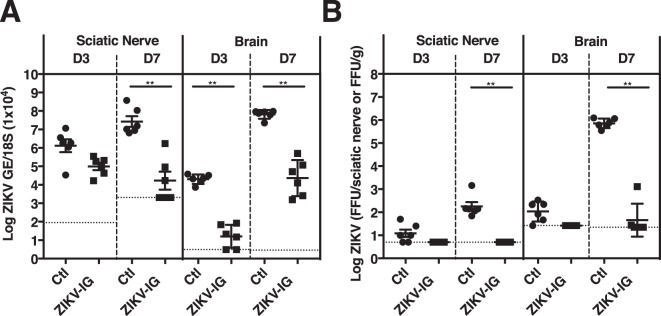
ZIKV-IG treatment decreases viral burden in the sciatic nerve and brain of ZIKV-infected mice. Groups of *Ifnar1*^−/−^ mice (n = 6) were infected with 1.0 × 10^3^ FFU of ZIKV strain FSS13025 (via r.o. route). At 24 hrs p.i., mice were treated (r.o. route) with vehicle (circle) or 50 (square) mg/kg ZIKV-IG. (**A**) ZIKV RNA levels, as measured by qRT-PCR and (**B**) infectious ZIKV levels, as determined by FFA analyses in the sciatic nerve and brain at days 3 and 7 p.i. Dotted lines indicate the limit of detection. Study groups were compared for statistical significance using Bonferroni corrected non-parametric Wilcoxon Rank-Sum tests for each tissue and time-point. Mean and standard error of the mean are shown.

As the major consequences of ZIKV infection are related to infection of the nervous system, we next compared levels of viral RNA and BHK cell-infectious virus in the peripheral and central nervous system of ZIKV-IG-treated *vs*. control mice. At day 3 p.i., similar levels of ZIKV RNA and infectious particles were present in the sciatic nerves of control and ZIKV-IG-treated mice, but at day 7 p.i., both viral RNA (−2.6-fold, p = 0.009) and BHK cell-infectious ZIKV (−3.2-fold, p = 0.009) levels were reduced in the sciatic nerves of animals treated with 50 mg/kg ZIKV-IG relative to control mice (Fig. 3). Treatment with lower doses of ZIKV-IG also decreased levels of both viral RNA (2 mg/kg ZIKV-IG, −1.1-fold, p = 0.035) and BHK cell-infectious virus (10 mg/kg ZIKV-IG, −2.6-fold, p = 0.009) in the sciatic nerves at day 7 p.i (Supplementary Fig. S4 and Table S2). In the brain, 50 mg/kg ZIKV-IG treatment significantly reduced viral RNA levels at both days 3 and 7 p.i. (day 3 p.i. −4.1-fold, p = 0.009 and day 7 p.i. −1.8-fold, p = 0.009) relative to control mice (Fig. 3A and Supplementary Tables S2). Only low levels of BHK cell-infectious ZIKV were detected at day 3 p.i. in the brains of both 50 mg/kg ZIKV-IG and control groups. However, at day 7 p.i., control mouse brains carried high levels of BHK cell-infectious ZIKV, and the brains from 50 mg/kg ZIKV-IG-treated mice contained reduced levels of BHK cell-infectious ZIKV relative to control mice (−3.5-fold, p = 0.009) (Fig. 3B and Supplementary Table S2). Viral RNA (−1.3-fold, p = 0.009) and BHK cell-infectious ZIKV (−1.8-fold, p = 0.009) levels were also decreased the day 7 p.i. brains of mice treated with 10 mg/kg ZIKV-IG (Supplementary Fig. S4 and Table S2). These results are consistent with the sciatic nerves and brain being major target organs of ZIKV at a later time point post infection in this mouse model, and show that ZIKV-IG treatment reduces viral burden in both peripheral and central nervous tissues.

**Figure 4:**
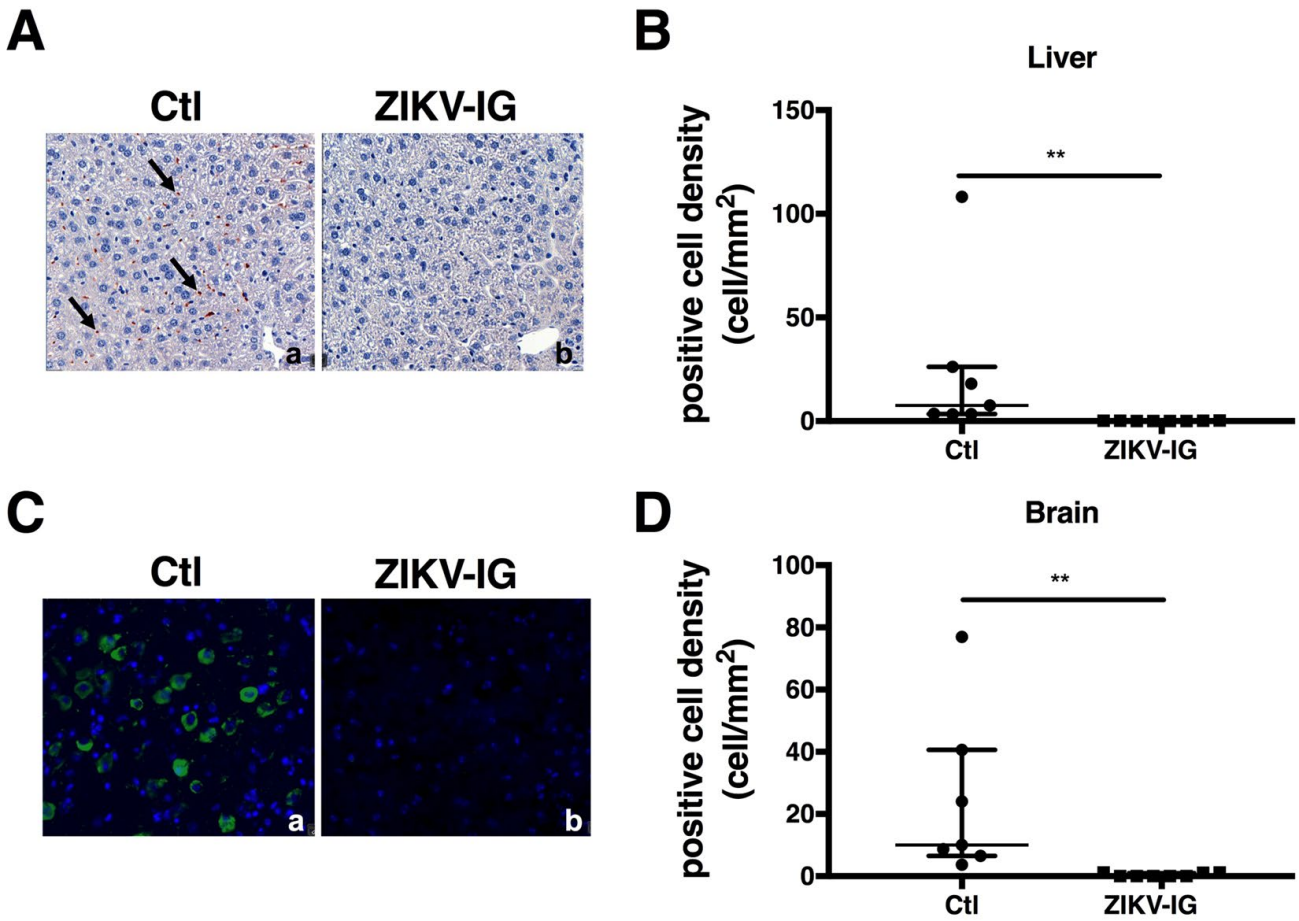
ZIKV-IG treatment decreases ZIKV NS2B expression in the liver and brain of ZIKV-infected mice. Groups of *Ifnar1*^−/−^ mice (n = 8) were infected with 1.0 × 10^3^ FFU of ZIKV strain FSS13025 (via r.o. route). At 24 hrs p.i., mice were treated (r.o. route) with vehicle or 50 mg/kg ZIKV-IG. At day 7 p.i., tissues were harvested to detect ZIKV NS2B expression via IHC. (**A**) NS2B expression in the liver (rust color dots, representative examples marked by arrows) of vehicle-treated mice after counterstaining with hematoxylin in cells morphologically consistent with LSECs and KCs. (**B**) NS2B expression in the liver was quantified using machine grading. (**C**) NS2B expression (green) was detected in vehicle-treated mice after counterstaining with DAPI in cells morphologically consistent with neurons and microglia. (**D**) NS2B expression was quantified using machine grading. Median and interquartile range are shown.

To confirm the robust efficacy of 50 mg/kg ZIKV-IG treatment against ZIKV infection in this mouse model, we next localized ZIKV in tissues by performing immunohistochemistry (IHC) for expression of ZIKV nonstructural protein 2B (NS2B), which is absent in virions and thus serves as a marker of viral replication. In the livers of control mice (Fig. 4Aa), NS2B was expressed in cells lining sinusoids that were morphologically consistent with liver sinusoidal endothelial cells and Kupffer cells (KC). In comparison, little to no NS2B immunoreactivity was seen in livers of mice treated with 50 mg/kg ZIKV-IG (Fig. 4Ab). Quantitative image analysis by ImageDx^TM^ software showed that median positive cell density in control mice was 7.55 cells/mm^2^ relative to 0.13 cells/mm^2^ in 50 mg/kg ZIKV-IG-treated mice (p = 0.006) (Fig. 4B). In the brain, NS2B was expressed in cells morphologically consistent with neurons and microglia in control mice (Fig. 4Ca), whereas little to no NS2B immunoreactivity was seen in mice treated with 50 mg/kg ZIKV-IG (Fig. 4Cb). Quantitative analysis showed the median positive cell density in control mice was 10.88 cells/mm^2^ as compared to 0.03 cells/mm^2^ in 50 mg/kg ZIKV-IG-treated mice (p = 0.006) (Fig. 4D). Thus, 50 mg/kg ZIKV-IG treatment is effective against ZIKV infection in this highly stringent challenge model, as assessed via three different assays (qRT-PCR, FFA, and IHC). In particular, these results show that 50 mg/kg ZIKV-IG treatment reduces viral burden in multiple tissues of *Ifnar1*^−/−^ mice at both the early (day 3) and late (day 7) time points after infection. In comparison to 50 mg/kg ZIKV-IG, lower doses of ZIKV-IG are less effective against ZIKV infection, reducing viral burden in select tissues.

### ZIKV-IG treatment decreases ZIKV-induced brain pathology

ZIKV’s neurotropism and associated brain pathology has been extensively documented in humans and *Ifnar1*^−/−^ mice. Therefore, to evaluate the extent of brain tissue pathology and other signs of injury in ZIKV-IG-treated and control mice following ZIKV infection, hematoxylin & eosin-stained brain slide sections were examined and scored by a blinded board-certified pathologist. Scores for these sections are shown in Table 3. The most significant and severe lesions seen in the brains of the control group included nonsuppurative encephalitis, gliosis, nonsuppurative meningitis, neuronal necrosis, and malacia (Table 3 and Supplementary Fig. S5). In contrast, little or no pathology was observed in mice treated with 50 mg/kg ZIKV-IG. Thus, treatment with 50 mg/kg ZIKV-IG reduces ZIKV-induced brain pathology (Table 3 and Supplementary Fig. S5).

**Table 3:** Grading of brain histopathology.

	Treatment Group	Grading score for brain lesion per mouse (0 to +++)								Most frequent observed severity (average severity)
		m1	m2	m3	m4	m5	m6	m7	m8	
**Nonsuppurative encephalitis**	Ctl	++	++	++	++	+++	+++	+++	N/A	++ (2.43)
	50 mg/kg	0	0	0	0	0	+	+	+	0 (0.38)
**Gliosis**	Ctl	+	++	++	++	++	++	+++	N/A	++ (2.00)
	50 mg/kg	0	0	0	0	+	+	+	+	0/+ (0.50)
**Nonsuppurative meningitis**	Ctl	+	++	++	++	++	++	++	N/A	++ (1.86)
	50 mg/kg	0	0	0	0	0	0	0	+	0 (0.13)
**Neural necrosis**	Ctl	+	+	+	+	++	++	++	N/A	+ (1.43)
	50 mg/kg	0	0	0	0	0	+	+	+	0 (0.38)
**Malacia**	Ctl	0	0	+	+	+	+	+	N/A	+ (0.71)
	50 mg/kg	0	0	0	0	0	0	0	+	0 (0.13)
**Hemorrhage**	Ctl	0	0	0	0	0	+	+	N/A	0 (0)
	50 mg/kg	0	0	0	0	0	0	0	0	0 (0)

## Discussion

In this study, we evaluated the therapeutic potential of human anti-ZIKV pAb (ZIKV-IG) against lethal ZIKV infection in a highly stringent mouse model. ZIKV-IG treatment was effective in protecting mice against ZIKV-induced mortality and morbidity by decreasing viral replication and dissemination into key target organs and ZIKV-induced pathology in the brain. These findings support further development of ZIKV-IG as a candidate for prophylaxis or treatment of Zika disease.

A variety of Abs, including mAbs isolated from ZIKV-immune individuals^62–64^ and human polyclonal antibody produced in transchromosomal bovines^65^ were shown to have therapeutic potential against ZIKV infection in mice. Similarly, transfer of convalescent sera from a human to pregnant mice prevented ZIKV infection and associated fetal birth defects^66^, and convalescent human plasma or sera obtained from ZIKV-infected individuals were able to neutralize both African and Asian ZIKV strains *in vitro*^67^. Here, we report that a single administration of 50 mg/kg human polyclonal Ab (ZIKV-IG) given at 24 hrs post-infection in *Ifnar1*^−/−^ mouse model of lethal ZIKV infection prevented both severe disease development and mortality. In addition, ZIKV-IG reduced ZIKV burden and ZIKV-induced tissue damage in target organs, confirming therapeutic potential against ZIKV infection. Recent outbreaks of several emerging and re-emerging viral diseases for which no approved treatment or vaccine exists have rekindled interest in the development of plasma-derived immunoglobulin therapeutic products. In our current study, we used human polyclonal IgG antibodies purified from convalescent donor plasma containing high titers of anti-ZIKV Abs. Plasma collection was based on criteria set by FDA and Emergent’s standards for virus screening and donor qualification. The immunoglobulin fraction was purified using a validated hyperimmune platform manufacturing process^68^.

Although, ZIKV strains have been phylogenetically characterized into African and Asian/American lineages, the virus has very little genome variability and is classified as a single serotype^67^. This premise is supported by the report that primary infection with ZIKV African strain in macaques protected the animals from secondary heterologous re-challenge with ZIKV Asian strain^69^. Thus, an effective ZIKV therapeutic candidate could potentially neutralize infection with any of the ZIKV virus lineages. Our *in vitro* potency based on anti-ZIKV neutralization titer and *in vivo* mouse results provide evidence that ZIKV-IG can effectively neutralize ZIKV infection.

Development of Ab products intended for use as a therapy against ZIKV infection should consider the risk of antibody dependent enhancement (ADE) of infection, which has previously been described for dengue virus (DENV), another member of the family *Flaviviridae*^70–75^. During the ADE process, pre-existing non- or sub-neutralizing Abs that recognize DENV enhance subsequent DENV infection and pathogenesis^76–78^. ZIKV is antigenically and genetically similar to DENV with ~56% genome sequence homology^79^, with *in vitro* and *in vivo* mouse studies demonstrating that Ab response to DENV and ZIKV can cross-react and cross-enhance infection and pathogenesis of each virus^79–83^. Although recent macaque and mouse studies have provided further support for pre-existing ZIKV Ab-mediated enhancement of subsequent DENV infection and disease severity^84–86^, passive transfer of vaccine-induced Abs before ZIKV challenge did not result in ZIKV infection enhancement or disease in non-pregnant mice and monkeys^87,88^. Consistent with these studies^84–88^, treatment with various sub-protective doses of ZIKV-IG showed no evidence for ADE of ZIKV infection in our mouse model as suggested by both survival and viral RNA results. No increase in mortality or viral burden were observed even using low ZIKV-IG concentrations which are potentially sub-neutralizing. However, viral load data obtained through focus forming assays using BHK cells may not be appropriate for drawing conclusions around the presence or absence of ADE as BHK cells lack expression of Fcγ receptors that support the ADE mode of infection.

Whether ADE is clinically relevant to human Zika disease is currently unknown, and thus, the possibility of ZIKV-IG-mediated ADE for Zika clinical disease remains a theoretical question for the development of Ab therapies against ZIKV. Further studies should be performed to assess whether ZIKV-IG has the potential to enhance DENV infection when given under pre-exposure setting.

In summary, we report that a single administration of 50 mg/kg of ZIKV-IG 24 hrs after infection protected mice against lethal ZIKV infection. Non-protective doses of ZIKV-IG did not induce ADE of ZIKV infection. These results provide the evidence that, at appropriate doses, ZIKV-IG treatment could be effective at preventing the deleterious effects of ZIKV in humans. Therefore, further testing in relevant pregnancy models to determine the impact of treatment on maternal and fetal infection is warranted.

## Material and Methods

Key reagents, Abs, primers, and probes used in this study are outlined in Supplementary Table S3.

### Virus

ZIKV strains MR766, FSS13025, and PRVABC59 were obtained from the World Reference Center for Emerging Viruses and Arboviruses (WRCEVA). FSS13025 is an Asian lineage strain isolated in 2010 from a pediatric case^89^. PRVABC59 is an Asian lineage strain isolated in 2015 from the blood of a human in Puerto Rico^90^. MR766 is an African lineage strain isolated in 1947 from a sentinel rhesus monkey (766) in Uganda^46^. Viruses were cultured using C6/36 *Aedes albopictus* mosquito cells, as described previously^91,92^. Virus was titrated using a baby hamster kidney (BHK)-21 cell-based focus-forming assay (FFA) as described previously^52^.

### Antibody neutralization assays

Naïve-ZIKV-IG and ZIKV-IG were initially diluted to 0.1 mg/mL and then serially diluted 1:3 for 11 dilutions in RPMI 1640 medium supplemented with 1% Hepes and 1% penicillin/streptomycin. Antibody dilutions were incubated at 37 °C with 5% CO_2_ for 1 hr with either 1 × 10^5^ FFU of ZIKV strain FSS13025, 2 × 10^4^ FFU of ZIKV strain MR766, or 1 × 10^4^ FFU of ZIKV strain PRVABC59. Standard flow cytometry-based neutralization assays using U937-DC-SIGN cells were then performed as described previously^58,86,93^.

### Mice and lethal ZIKV infection model

*Ifnar1*^−/−^ mice (C57BL/6 mice deficient in type I interferon receptor) were originally obtained from Dr. W. Yokoyama (Washington University, St. Louis. MO) and subsequently bred under specific pathogen-free conditions at the animal facility of La Jolla Institute for Immunology. All experiments were approved by the Institutional Animal Care and Use Committee under protocol number AP028-SS1-0615/AP00001029. All experiments included age- and sex-matched mice. At 5–6 weeks of age, mice were randomized at first by gender and secondly per weight. Mice were inoculated intravenously *via* the retro-orbital (r.o.) route with 1.0 × 10^3^ FFU of ZIKV FSS13025.

### Testing of drug product and treatments

ZIKV-IG was a purified human IgG product manufactured using plasma collected from US FDA licensed plasma centers screened for Ab reactive to ZIKV. Established processes^68^ were employed for manufacturing of ZIKV-IG (lot PD_740_ZKP_16_001_003_ER_v1) used in this study. This lot contained a total protein concentration of 54 mg/mL (>98.9% human IgG). Potency was determined using a microneutralization assay that measured the cytopathic effect of ZIKV strain PRVABC59 on Vero E6 cells using an xCELLigence® real-time cell analyzer (RTCA, ACEA Biosciences Inc.). Briefly, Vero E6 cells were added to an xCELLigence 96-well electronic microtiter plate (E-plate) then pre-incubated overnight at 37 °C in a humidified 5% CO_2_ incubator. The E-plate contains electrodes in each well; adherent cells in the wells impede the electric current passing through the electrodes. An equal volume of ZIKV PRVABC59, diluted to 100 TCID_50_, was incubated for one hour at 37 °C with serial dilutions of ZIKV-IG, then added to the E-plate. The E-plate was then incubated at 37 °C in an xCELLigence unit contained in a humidified 5% CO_2_ incubator. Cells were monitored in real-time by measuring ZIKV-induced changes in cell impedance at 30-minute intervals. Sample dilution data from the defined end point was analyzed by fitting the impedance measure (cell index) to the log dilution using a 4-parameter logistic curve fit to determine the 50% neutralizing titer (NT_50_). The initial potency value for ZIKV-IG product was 18,480, indicating a high degree of Zika virus neutralization.

Naïve-ZIKV-IG was manufactured using the same processes as ZIKV-IG, with the exception that the source plasma used did not contain Ab reactive to ZIKV. Mice were injected via r.o. route with 50, 10, 2.0, 0.5 or 0.1 mg/kg ZIKV-IG (100 μL/mouse) at 24 hrs following lethal ZIKV infection.

### Clinical monitoring of mice and euthanasia criteria

Following infection, mice were weighed and observed for clinical signs and scored daily. Clinical scores were based on mouse appearance, mobility, and attitude on a 7-point scale (Table S3). Animals losing ≥20% body weight were humanely euthanized.

### Viral quantification by qRT-PCR and Focus Forming Assay

#### Sample collection

Viral quantification was conducted by qRT-PCR and by Focus Forming Assay (FFA) on serum, spleen, kidney, liver, sciatic nerve, and brain. Sera were collected after centrifugation (15,900 g for 15 min at 4 °C) of blood harvested by cardiac puncture into collection tubes (Sarstedt, #41.1500.005). Following mouse perfusion with PBS, tissues were harvested and stored either in tubes containing RNA Later (Invitrogen) for qRT-PCR or in pre-weighed tubes containing MEMα medium and steel beads (Qiagen, #69989) for FFA. Tissues were then homogenized in RTL buffer (Qiagen) + 1% beta mercaptoethanol for qRT-PCR and MEMα medium for FFA followed by clarification (centrifugation at 2,000 × g for 5 min).

#### Viral RNA quantification by qRT-PCR

RNA from serum and homogenized tissues were isolated using the QIAmp Viral RNA Mini Kit (Qiagen) and the RNeasy Mini Kit (Qiagen), respectively. ZIKV RNA levels in sera and tissues were quantified by qRT-PCR. Specific primers and probes used are listed in Table S2. Tissue RNA levels were normalized to 18S as described for Dengue RNA^91^ and expressed as genome equivalent per 18S (GE/18S), while serum RNA levels were expressed as GE/mL.

#### Quantification of infectious ZIKV by FFA

FFA procedures were performed as previously described^52^. Tissue samples were homogenized and clarified by centrifugation. Tissue supernatants and sera were diluted serially before infection on BHK cells. BHK cells used in this assay do not express Fcγ receptors. Cells were plated (2.0 × 10^5^ cells/well in a 24-well plate) and incubated overnight at 37 °C, 5% CO_2_. Confluent monolayers were inoculated with undiluted or 10-fold serially diluted sera or clarified tissue supernatant, and were incubated for 1 hr at 37 °C. After incubation, the inoculum was removed, and each cell monolayer was overlaid with CMC-media and incubated at 37 °C, 5% CO_2_ for 1.5–2 days. Cells were then fixed, permeabilized, and incubated with pan *Flavivirus* anti-envelope (E) Ab clone 4G2 (BioXCell), followed by incubation with horseradish peroxidase-conjugated goat anti-mouse IgG secondary antibody (Jackson ImmunoResearch) and staining with True-Blue peroxidase substrate (Sera Care). Foci were counted, virus levels in the serum were expressed as Focus Forming Units (FFU) per mL, and for most tissues as FFU/g. As it was not technically feasible to weigh sciatic nerves, viral levels in these tissues were expressed as FFU/tissue.

### Histopathology

At the time of necropsy, liver and brain were collected in 10% zinc formalin (BBC Biochemical) for histopathologic evaluation. Tissues were fixed for 48 hrs at room temperature. Brains were cut transversely at points corresponding to Bregma +2 mm and Bregma −3 mm of Mouse Brain Atlas celloidin case #170 (http://www.mbl.org), resulting in 3 brain sections (rostral, middle, and caudate). Tissues were processed routinely for paraffinization and cut at 4 μm thickness for H&E staining. Slides were blinded before review with an Olympus BX40 brightfield microscope at 2-60X magnification. Lesions were graded on a 4-point scale (0 to +++). All categories of lesions were tabulated (Table 3).

### Enzyme immunohistochemistry

Paraffinized liver was cut onto slides (4 μm) and routinely deparaffinized. Slides were microwaved (GE, Model#: JES1142WD04) on high setting in Antigen Unmasking Solution (Vector Laboratories) and cooled at room temperature. Endogenous enzyme activity was blocked with BLOXALL (Vector Laboratories) and nonspecific protein binding was blocked with 10% Normal Goat Serum (Thermo Fisher). Slides were incubated with anti-ZIKV NS2B Ab (Genetex) at 4 °C for 14 hrs. Slides were incubated with ImmPRESS HRP (Vector Laboratories) secondary Ab followed by incubation with ImmPACT NovaRED (Vector Laboratories) substrate. Slides were counterstained with Modified Mayer’s Hematoxylin (Thermo Fisher) and routinely processed for mounting. Positive, negative, and rabbit IgG (Vector Laboratories) controls were included with each batch. Morphology of immuno-reactive cells were confirmed by a board-certified pathologist, and immunoreactivity was quantified by ImageDx^TM^ (Reveal Biosciences).

### Immunofluorescence

At the time of necropsy, brains were collected in 4% paraformaldehyde (Alfa Aesar) at 4 °C and allowed to fix for 24 hrs. Tissues were then cryoprotected by serial immersion in 15% sucrose (Affymetrix) followed by 20% sucrose until the brains floated. Brains were cut transversely at points corresponding to Bregma +2 mm and Bregma −3 mm of Mouse Brain Atlas celloidin case #170 (http:/www.mbl.org). Tissues were transferred to cryomolds, embedded in OCT (Electron Microscopy Services), and frozen on dry ice followed by −80 °C freezer. Tissues were cut at 10 μm thickness for immunofluorescence. Slides were microwaved (GE, Model#: JES1142WD04) on high setting in Antigen Unmasking Solution (Vector Laboratories) and cooled at room temperature. Slides were incubated in Tris-Urea buffer and permeabilized with 0.1% Triton X-100 (Acros Organics). Nonspecific protein binding was blocked with 10% Horse Serum (Thermo Fisher). Slides were incubated with anti-ZIKV NS2B Ab (Genetex) at 4 °C for 14 hrs, followed by incubation with anti-rabbit AlexaFluor 488 (Invitrogen) secondary Ab and DAPI (Invitrogen) counterstain. Cover slips were mounted with Prolong Gold (Invitrogen). Positive, negative, and rabbit IgG (Vector Laboratories) controls were included with each batch. Morphology of cells in the brains was confirmed by a board-certified pathologist. Brain immunoreactivity was quantified by ImageDx^TM^ (Reveal Biosciences).

### Statistical analyses

Statistical analysis was performed using SAS version 9.3 statistical software (SAS). Survival rates and median time to death (MTD) were estimated using the Kaplan-Meier method. Data were analyzed either with Fisher’s exact test (survival), Log-rank test (MTD) or exact Wilcoxon rank-sum test (viral RNA and infectious virus). All these tests were followed by a Bonferroni correction. Immunohistochemistry and immunofluorescence data for both brain and liver were analyzed with Kruskal-Wallis rank-sum test with Dwass, Steel, Critchlow-Fligner correction for multiple comparisons. Results were considered significant when p < 0.05.

## Supplementary information

Supplementary file
