# Supplementary material for: Human Polyclonal Antibodies Prevent Lethal Zika Virus Infection in Mice
Source: Sci Rep. 2019 Jul 8;9:9857. doi: 10.1038/s41598-019-46291-9 (PMC6614477; doi:10.1038/s41598-019-46291-9)
Supplement: Supplementary file 1 — Supplementary file [file 41598_2019_46291_MOESM1_ESM.pdf]

Human Polyclonal Antibodies Prevent Lethal Zika Virus Infection in Mice

Emilie Branche, Ayo Yila Simon, Nicholas Sheets, Kenneth Kim, Douglas Barker, Anh-Viet T. Nguyen, Harpreet Sahota, Matthew Perry Young, Rebecca Salgado, Anila Mamidi, Karla M. Viramontes, Trevor Carnelley, Hongyu Qiu, Annie Elong Ngono, Jose Angel Regla-Nava, Mercylia Xevana Susantono, Joan M. Valls Cuevas, Kieron Kennedy, Shantha Kodihalli and Sujan Shresta

Supplementary information:

- **Supplementary Figures:**

FIGURE S1

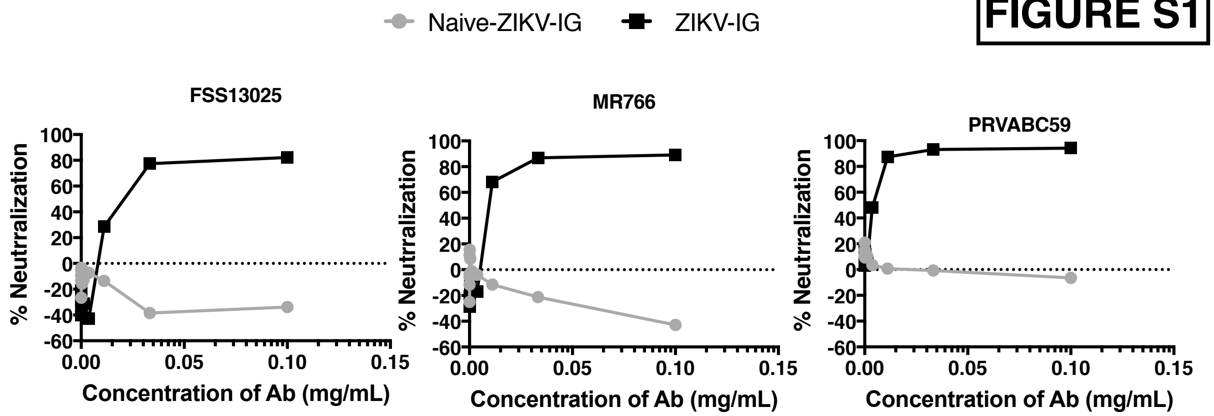

FIGURE S2

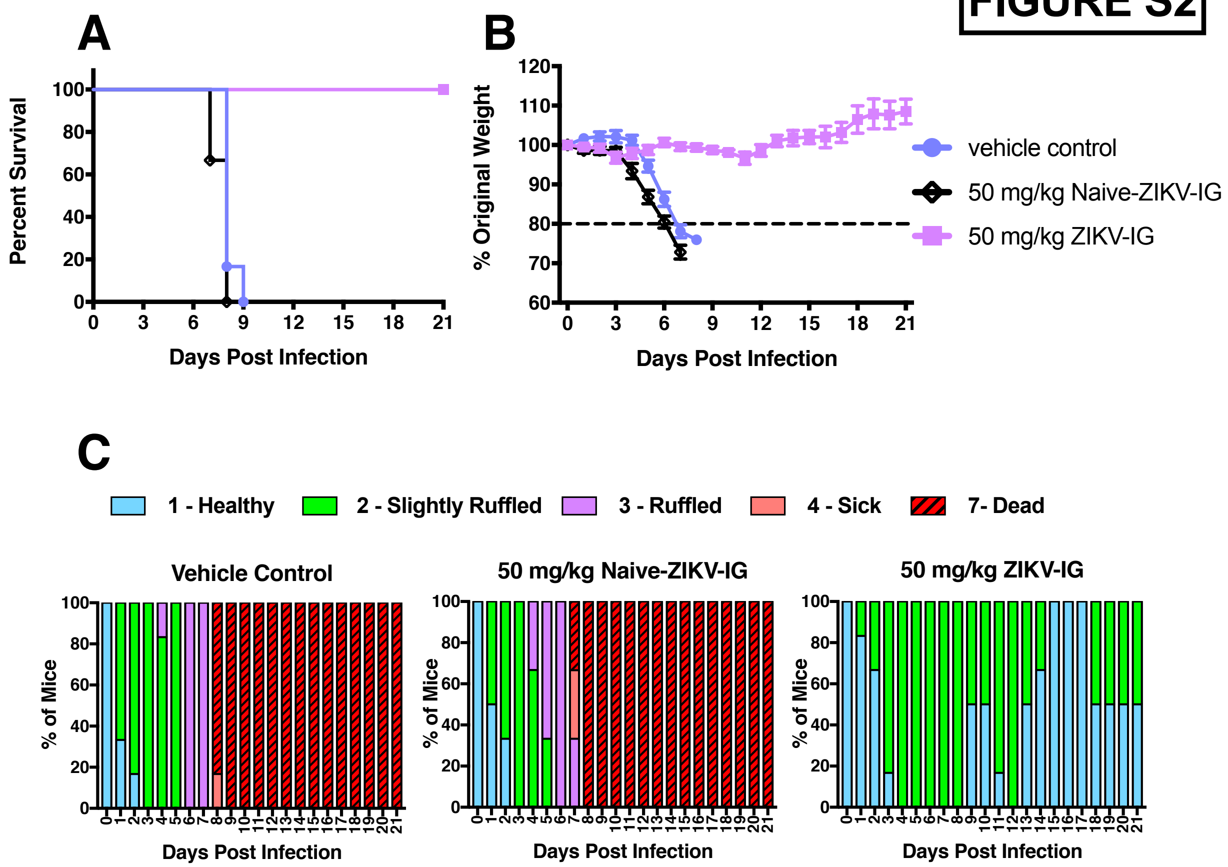

Figure S3

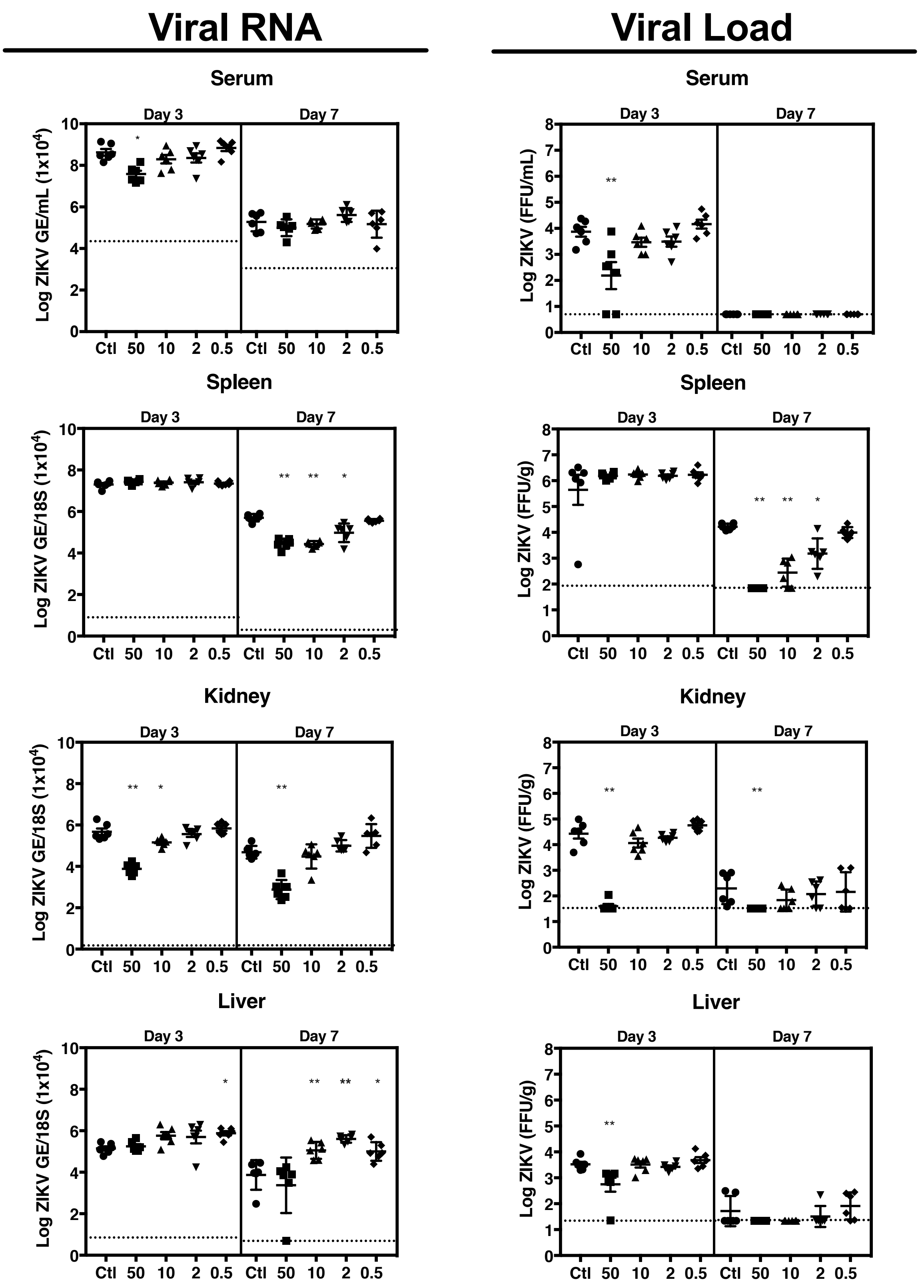
Viral RNA
Viral Load
Serum
Spleen
Kidney
Liver
Day 3
Day 7
Log ZIKV GE/mL (1x10⁴)
Log ZIKV GE/18S (1x10⁴)
Log ZIKV (FFU/mL)
Log ZIKV (FFU/g)
Ctl 50 10 2 0.5 Ctl 50 10 2 0.5

Figure S4

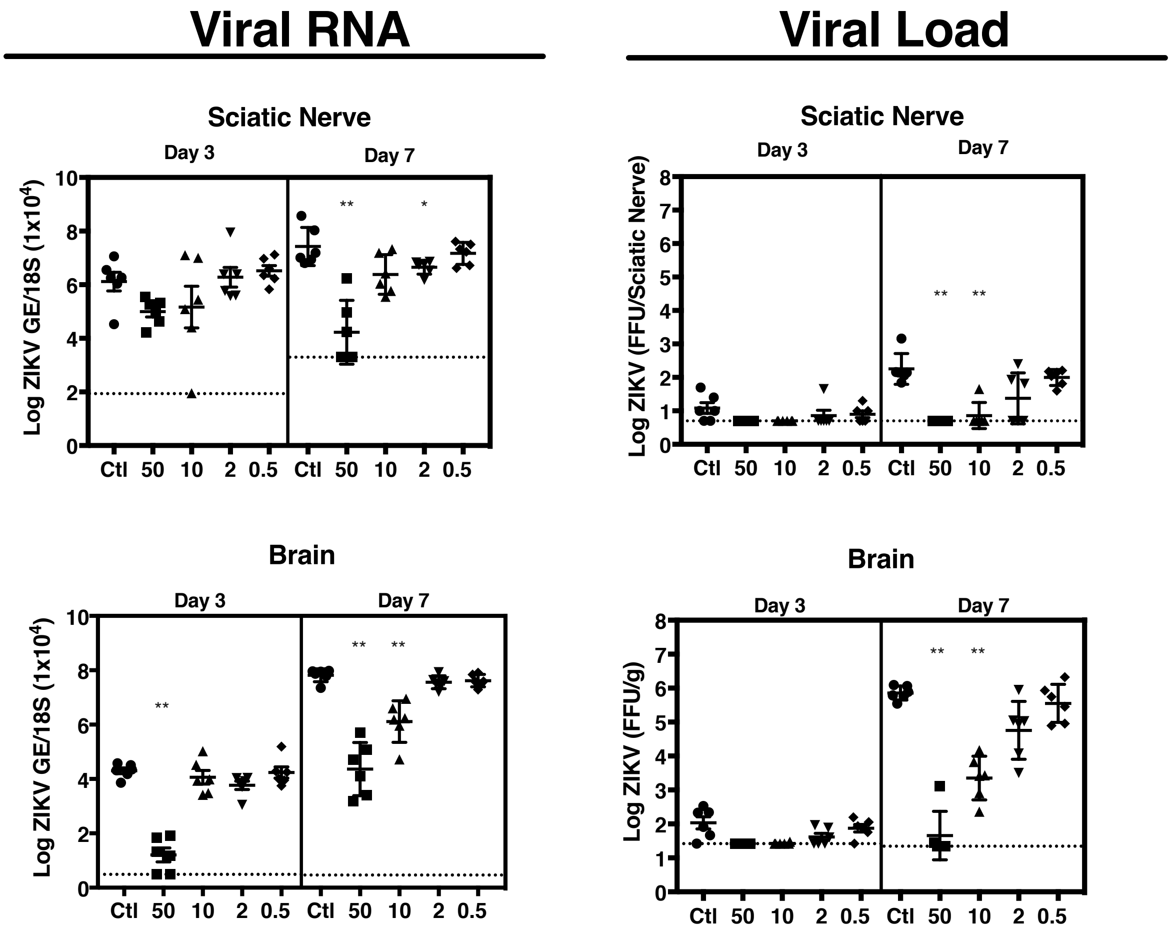

Viral RNA
Viral Load
Sciatic Nerve
Day 3
Day 7
Log ZIKV GE/18S (1x10^4)
Log ZIKV (FFU/Sciatic Nerve)
Brain
Log ZIKV (FFU/g)
Ctl 50 10 2 0.5 Ctl 50 10 2 0.5
**
*

FIGURE S5

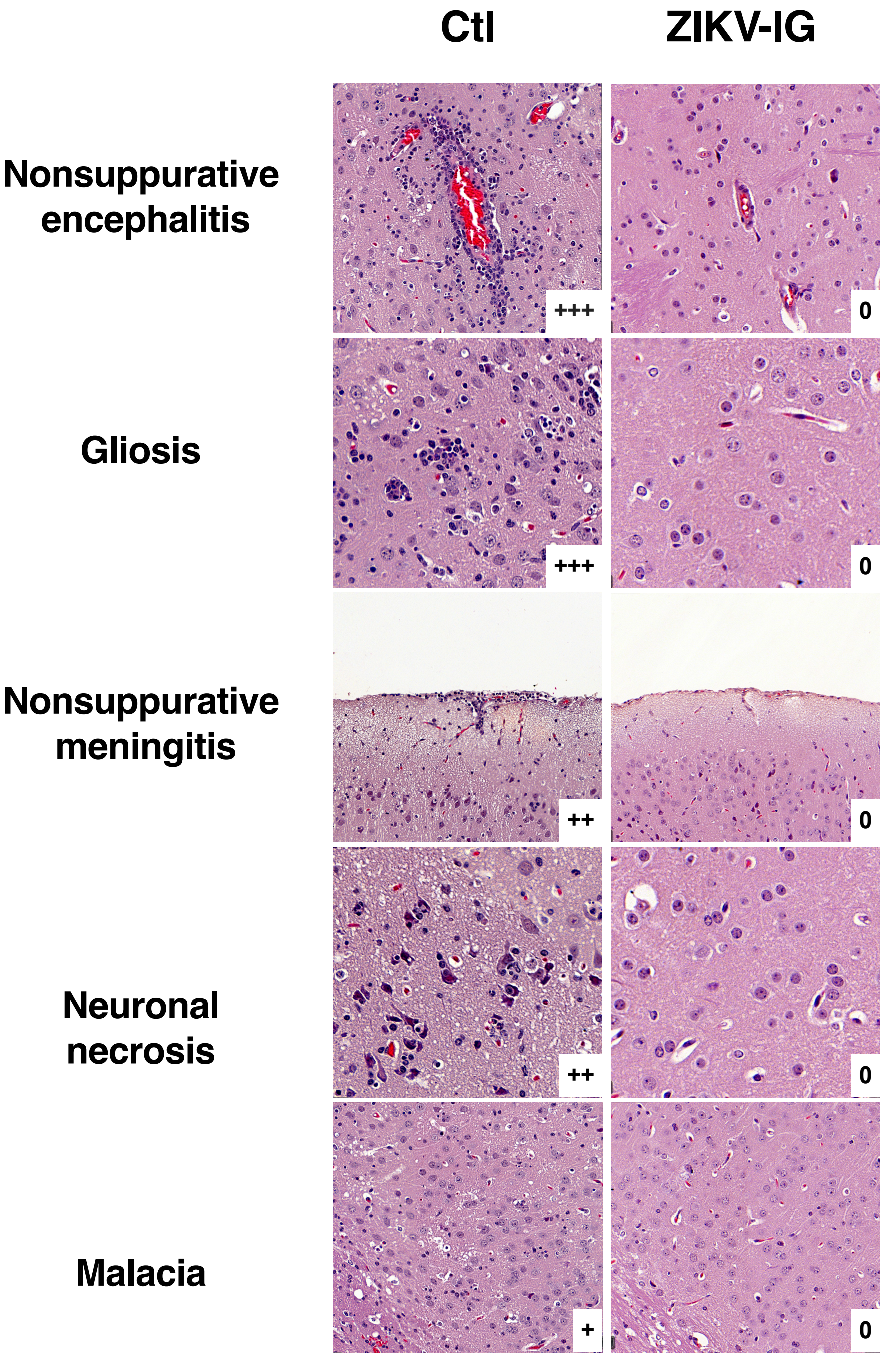
Ctl
ZIKV-IG
Nonsuppurative encephalitis
+++
0
Gliosis
+++
0
Nonsuppurative meningitis
++
0
Neuronal necrosis
++
0
Malacia
+
0

Supplementary Legends

Figure S1: ZIKV-IG neutralized FSS13025, MR766 and PRVABC59 ZIKV strains in vitro

Neutralization capacity of ZIKV-IG and placebo (naïve-ZIKV-IG) was assessed against ZIKV strains FSS13025, MR766, and PRVABC59 via U937-DC-SIGN cells and flow cytometry-based assays. Dotted line indicates 0% neutralization. ZIKV-IG (black squares) was compared to naïve-ZIKV-IG (grey circles).

Figure S2: Both Vehicle control and naïve-ZIKV-IG treatments do not enhance survival or reduce weight loss in ZIKV infected *Ifnar1*$^{-/-}$ mice

Groups of *Ifnar1*$^{-/-}$ mice (n=6) were infected with 1.0 x 10^3 FFU of ZIKV strain FSS13025 by the retro-orbital (r.o.) route. At 24 hrs p.i., mice were treated (via the r.o. route) with vehicle, 50 mg/kg naïve-ZIKV-IG or 50 mg-kg ZIKV-IG. (**A**) Kaplan–Meier survival curves. (**B**) Mean percent weights, which are plotted for each group relative to the percent weight on day 0 (baseline). (**C**) Clinical scores. Error bars represent standard error of the mean.

Figure S3: ZIKV-IG treatment dose effects on viral RNA and infectious virus levels in the serum, spleen, kidney and liver of ZIKV infected-mice

Groups of *Ifnar1*$^{-/-}$ mice (n=6) were infected with 1.0 x 10^3 FFU of ZIKV FSS13025 (via r.o.route). At 24 hrs p.i., mice were treated (r.o. route) with vehicle (circle), 50 (square), 10 (triangle point up), 2 (triangle point down) or 0.5 (diamond) mg/kg of ZIKV-IG. Viral RNA levels were determined by qRT-PCR (left panel) and infectious virus level by FFA (right panel) in the serum, kidney, spleen and liver at days 3 and 7 p.i. Dotted lines indicate the limit of detection. Treated groups were compared to vehicle control group for each tissue type and time-point using

Bonferroni corrected non-parametric Wilcoxon Rank-Sum tests. Error bars represent standard error of the mean.

Figure S4: ZIKV-IG treatment dose effects on viral RNA and infectious virus levels in the sciatic nerve and brain of ZIKV infected-mice

Groups of *Ifnar1*$^{-/-}$ mice (n=6) were infected with 1.0 x 10^3 FFU of ZIKV FSS13025 (r.o. route). At 24 hrs, mice were treated (r.o. route) with vehicle, (circle), 50 (square), 10 (triangle point up), 2 (triangle point down) or 0.5 (diamond) mg/kg of ZIKV-IG. Viral RNA levels were determined by qRT-PCR (left panel) and infectious virus levels by FFA (right panel) in the sciatic nerves and brain at days 3 and 7 p.i. Dotted lines indicate the limit of detection. Groups were performed for each tissue type and time-point using Bonferroni corrected non-parametric Wilcoxon Rank-Sum tests. Error bars represent standard error of the mean.

Figure S5: ZIKV-IG treatment decreases severity of meningoencephalitis in ZIKV infected-mice

Groups of *Ifnar1*$^{-/-}$ mice (n=8) were infected with 1.0 x 10^3 FFU of ZIKV FSS13025 (r.o. route). At 24 hrs p.i., mice were treated (r.o. route) with vehicle control or 50 mg/kg of ZIKV-IG. In vehicle-treated control mice (left column), pathologic changes included nonsuppurative encephalitis, gliosis, nonsuppurative meningitis, neuronal necrosis, and malacia. These lesions were minimal to absent in mice treated with 50mg/kg ZIKV-IG (right column). Grades of lesions (0 to +++) are shown with each corresponding micrograph. Hematoxylin & eosin stain.

- **Supplementary Tables:**

Table 2: Analysis of median time to death between vehicle control group naïve-ZIKV-IG and ZIKV-IG-treated groups

Treatment Group	% survival	Fisher Exact Test p-value	Median Survival (days)	Logrank Test p-value
	**Comparison to vehicle control**			
Vehicle control	0	NA	8	NA
50 mg/kg naïve ZIKV-IG	0	> 0.9999	8	0.0930
50 mg/kg ZIKV-IG	100	0.0022**	Undefined	0.0005**
	**Comparison to naïve-ZIKV-IG**			
Vehicle control	0	> 0.9999	8	0.0930
50 mg/kg naïve ZIKV-IG	0	NA	8	NA
50 mg/kg ZIKV-IG	100	0.0022**	Undefined	0.0012**

** statistically significant ($p < 0.05$)

Table S2: Statistical analysis results of ZIKV-IG therapeutic dose effect as compared to vehicle control on viral RNA and infectious virus levels

		**Viral RNA levels based on qRT-PCR**				**Infectious virus levels based on FFA**			
		**Day 3**		**Day7**		**Day 3**		**Day7**	
Tissue	ZIKV-IG dose (mg/kg)	Exact Wilcoxon rank-sum p-value vs vehicle control	Bonferroni adjusted p-value vs vehicle control	Exact Wilcoxon rank-sum p-value vs vehicle control	Bonferroni adjusted p-value vs vehicle control	Exact Wilcoxon rank-sum p-value vs vehicle control	Bonferroni adjusted p-value vs vehicle control	Exact Wilcoxon rank-sum p-value vs vehicle control	Bonferroni adjusted p-value vs vehicle control
Serum	50	0.004*	0.017*	0.310	1.000	0.011*	0.043*	1.000	1.000
	10	0.310	1.000	0.818	1.000	0.169	0.675	1.000	1.000
	2	0.485	1.000	0.180	0.719	0.240	0.961	1.000	1.000
	0.5	0.394	1.000	1.000	1.000	0.394	1.000	1.000	1.000
Spleen	50	0.180	0.719	0.002*	0.009*	1.000	1.000	0.002*	0.009*
	10	0.485	1.000	0.002*	0.009*	0.937	1.000	0.002*	0.009*
	2	0.310	1.000	0.004*	0.017*	0.937	1.000	0.009*	0.035*
	0.5	0.937	1.000	0.065	0.260	0.699	1.000	0.065	0.260
Kidney	50	0.002*	0.009*	0.002*	0.009*	0.002*	0.009*	0.002*	0.009*

	10	0.009*	0.035*	0.937	1.000	0.180	0.719	0.128	0.511
	2	0.937	1.000	0.093	0.372	0.394	1.000	0.370	1.000
	0.5	0.240	0.961	0.015*	0.061	0.240	0.961	0.660	1.000
Liver	50	0.485	1.000	0.310	1.000	0.002*	0.009*	0.455	1.000
	10	0.026*	0.104	0.002*	0.009*	0.818	1.000	0.455	1.000
	2	0.065	0.260	0.002*	0.009*	0.589	1.000	0.455	1.000
	0.5	0.004*	0.017*	0.004*	0.017*	0.310	1.000	0.275	1.000
Sciatic Nerve	50	0.041*	0.165	0.002*	0.009*	0.061	0.242	0.002*	0.009*
	10	0.485	1.000	0.093	0.372	0.061	0.242	0.002*	0.009*
	2	0.937	1.000	0.009*	0.035*	0.197	0.788	0.054	0.216
	0.5	0.485	1.000	0.818	1.000	0.448	1.000	0.584	1.000
Brain	50	0.002*	0.009*	0.002*	0.009*	0.015*	0.061	0.002*	0.009*
	10	0.485	1.000	0.002*	0.009*	0.015*	0.061	0.002*	0.009*
	2	0.015*	0.061	0.093	0.372	0.102	0.407	0.026*	0.104
	0.5	0.394	1.000	0.132	0.528	0.515	1.000	0.310	1.000

* =Significant Wilcoxon rank-sum p-value ≤ 0.05 (Bonferroni adjusted p-value ≤ 0.05).

Table S3: Reagents, antibodies, primers, and probes used in this study

Real-time PCR primers

Name	Forward	Reverse
ZIKV	5′-TTGGTCATGATACTGCTGATTGC-3’	5′-CCTTCCACAAAGTCCCTATTGC-3′
18S	5′-CGGCTACCACATCCAAGGAA-3′	5′-GCTGGAATTACCGCGGCT-3′

Real-time PCR probes

Name	Probe
ZIKA	5′-[6-FAM]-CGGCATACAGCATCAGGTGCATAGGAG-[Tamra-Q]-3′
18S	5′-[6-FAM]-TGCTGGCACCAGACTTGCCCTC-[Tamra-Q]-3′

Antibodies

Name/Protein targeted	Clone	Provider	Catalog number
mouse pan-flavivirus	4G2	BioXcell	hybridoma from ATCC
Peroxidase AffiniPure Goat Anti-Mouse IgG antibody		Jackson	115-035-072
Antibodies FOR IF AND IHC	ZIKV NS2B Polyclonal	Genetex	GTX133308
ZIKV-IG		Emergent BioSolutions Canada Inc.	Lot # PD_740_ZKP_16_001_003_ER_vl
Naive-ZIKV-IG		Emergent BioSolutions Canada Inc.	lot# : PD_740_16_001_007

PE-labeled anti-human CD209		BD Pharmingen	551265

Other reagents

Name	Provider	Catalog number
QIAmp Viral RNA Mini Kit	Qiagen	52906
RNeasy Mini Kit	Qiagen	74106
qScript One-Step qRT-PCR kit	Quanta Biosciences	95057-200
RNAlater	Invitrogen	AM7021
RLT buffer	Qiagen	79216
True blue	Sera Care	5510-0030
Carboyxmethyl Cellulose Sodium Salt, Medium Viscosity (CMC)	Sigma	C9481-500G
True-Blue	SERA CARE	5510-0030
10% Buffered Formalin Phosphate	Fischer Chemical	SF100-4
10% Zinc Formalin	BBC Biochemical	MA0102414
Sucrose	Affymetrix	MFCD00006626
Formic Acid 88%	Fisher Chemical	MFCD00003297
Tissue-Tek OCT	Electron Microscopy Services	6255001
Paraformaldehyde 16%	Alfa Aesar	3025-89-4
Horse serum	Vector Laboratories	S-2000
Rabbit IgG	Vector Laboratories	I-1000
Donkey anti-rabbit AlexaFluor 488	Thermo Fisher	A-21206
DAPI	Invitrogen	D1306
Antigen Unmasking Solution	Vector Laboratories	H-3300
BLOXALL	Vector Laboratories	SP-6000
Normal goat serum	Thermo Fisher	50062Z

ImmPRESS HRP	Vector Laboratories	MP-7451
ImmPACT NovaRED	Vector Laboratories	SK-4805
Modified Mayer's Hematoxylin	Thermo Fisher	72804
RPMI 1640	Gibco	11875-093
HEPES	Gibco	15630-080
penicillin/streptomycin	Gibco	15140-122
Cytofix/Cytoperm	BD Bioscience,	51-2090ZK
10x Perm/Wash Buffer	BD Bioscience	51-2091KZ
Pierce FITC using an antibody labelling kit	Thermo Fisher Scientific	53027

Table S4: Clinical signs score on a 7-point scale

SCORE	INITIALS	DESCRIPTION	APPEARANCE	MOBILITY	ATTITUDE
**1**	H	Healthy	Smooth Coat. Bright Eyes.	Active, Scurrying, Burrowing	Alert
**2**	SR	Slightly Ruffled	Slightly Ruffled coat (usually only around head and neck)	Active, Scurrying, Burrowing	Alert
**3**	R	Ruffled	Ruffled Coat throughout body. A “wet” appearance.	Active, Scurrying, Burrowing	Alert
**4**	S	Sick	Very Ruffled coat. Slightly closed, inset eyes.	Walking, but no scurrying.	Mildly Lethargic
**5**	VS	Very Sick	Very Ruffled Coat. Closed, inset eyes.	Slow to no movement. Will return to upright position if put on its side.	Extremely Lethargic
**6**	E	Euthanize	Very ruffled Coat. Closed, inset eyes. Moribund requiring humane euthanasia.	No movement or uncontrollable, spastic movements. Will NOT return to upright position if put on its side.	Completely Unaware or in Noticeable Distress
**7**	D	Deceased	---	---	---
